# Gender-dependent effects of gonadectomy on lung carcinogenesis by 4-(methylnitrosamino)-1-(3-pyridyl)-1-butanone (NNK) in female and male A/J mice

**DOI:** 10.3892/or.2013.2759

**Published:** 2013-10-01

**Authors:** FUMIKO NINOMIYA, MASANAO YOKOHIRA, SOSUKE KISHI, YUKO NAKANO, KEIKO YAMAKAWA, TATSUSHI INOUE, TOSHIYA KUNO, KATSUMI IMAIDA

**Affiliations:** Onco-Pathology, Department of Pathology and Host-Defense, Faculty of Medicine, Kagawa University, Miki-cho, Kita-gun, Kagawa 761-0793, Japan

**Keywords:** lung tumorigenesis, gonadectomy, sex hormone, 4-(methylnitrosamino)-1-(3-pyridyl)-1-butanone, A/J mouse

## Abstract

The present study was conducted to investigate the effects of gonadectomy on lung carcinogenesis in female and male mice, and to determine an association between sex hormone and lung carcinogenesis. Female and male A/J mice were divided into gonadectomized and unoperated control groups and all animals were treated intraperitoneally with 1 or 2 injections of 4-(methylnitrosamino)-1-(3-pyridyl)-1-butanone (NNK) at the dose of 2 mg/mouse. The mice were sacrificed 18 or 56 weeks after surgery. Serum levels of estradiol in females and testosterone in males were confirmed to be decreased by gonadectomy. Lung white nodules were detected in all mice of all groups. In the control groups of 18- and 56-week studies, the multiplicities of lung nodules in females were significantly greater than in males. In males in the 56-week study, the multiplicity of macroscopical lung nodules, bronchiolo-alveolar hyperplasias, adenomas and tumors (adenomas and adenocarcinomas) showed significant increase with castration. In females in the 18-week study, the multiplicity of adenomas decreased significantly by ovariectomy. Based on the results of the present study, female A/J mice were confirmed to be more susceptible to NNK-induced lung carcinogenesis than males. Furthermore, it was suggested that the process is inhibited by testosterone and accelerated by estradiol. These findings indicate the possibility that sex hormones play important roles in determining sex differences in lung carcinogenesis in the A/J mice initiated by NNK.

## Introduction

Lung cancer prognosis remains very poor, with a 5-year survival rate generally <20%, lower than several other leading cancers, underlining the need for a better understanding of lung cancer formation and, ultimately, for identification of better therapeutic targets (1). Cigarette smoking is acknowledged to be the most important risk factor for human lung carcinogenesis and 4-(methylnitrosamino)-1-(3-pyridyl)-1-butanone (NNK) is a tobacco-specific N-nitrosamine which is considered to play important roles in tobacco-related human lung cancer (2,3). NNK is also a strong lung carcinogen in rodents, inducing bronchiolo-alveolar hyperplasia, adenoma and adenocarcinoma (4). Previously, we demonstrated that NNK-induced lung carcinogenesis in female A/J mice was strongly inhibited by treatment with 8-methoxypsoralen, a potent human cytochrome P450 2A6 (CYP2A6) inhibitor, before a single intraperitoneal injection (i.p.) of NNK (5–9). We also reported carcinogenic mechanisms and modifying effects of other factors using NNK-induced lung cancer animal models (10–15).

In the mouse, it is well known that females are generally more sensitive to chemical lung carcinogenesis, such as that induced by NNK, than males (16). It is hypothesized that the difference is due to difference in the amount of sex hormones such as testosterone and estradiol. Also, in humans, the incidence of adenocarcinoma type lung cancer is known to be generally higher in women than in men (17–19). The available data suggest that gender and sex hormones affect the character of lung cancer (20,21). For the purpose of these experiments, we hypothesized that the carcinogenesis of the lung may be influenced by sex hormone levels.

The present study was conducted to investigate effects of gonadectomy, ovariectomy and castration in female and male mice, respectively, on lung carcinogenesis and to determine associations with sex hormone levels, i.e. estradiol and testosterone. The experiments were conducted for 16 and 54 weeks after NNK treatment, since hyperplasia and adenoma lesions were earlier detected after 16 weeks (9), and adenocarcinomas were detected after 54 weeks (6).

## Materials and methods

### Chemicals

NNK was purchased from Toronto Research Chemicals (Toronto, Ontario, Canada).

### Animals

A total of 72 female and 72 male A/J mice (5 weeks of age), purchased from Shizuoka Laboratory Animal Center (Shizuoka, Japan), were maintained in the Division of Animal Experiments, Life Science Research Center, Kagawa University, according to the Institutional Regulations for Animal Experiments. The regulations included the best considerations on animal welfare and good practice of animal handling contributing to the replacement, refinement and reduction of animal testing (3Rs). The protocols of the experiments were approved by the Animal Care and Use Committee for Kagawa University. All animals were housed in polycarbonate cages with white wood chips for bedding, and given free access to drinking water and a basal diet, Oriental MF (Oriental Yeast Co., Ltd., Tokyo, Japan), under controlled conditions of humidity (60±10%), lighting (12 h light/dark cycle) and temperature (24±2°C).

### Experimental design

At 6 weeks of age, the mice were separated into 8 groups (Groups 1–8) of 15–21 animals each (Table I). Groups 1 and 5 were female groups undergoing ovariectomy. Groups 2 and 6 were females without ovariectomy. Groups 3 and 7 were male groups with castration, while Groups 4 and 8 were males without surgery. At the operations, the ovaries or the testes were removed under deep anesthesia (Groups 1, 3, 5 and 7) with i.p. of 0.025 ml pentobarbital sodium (Somunopentyl; Kyoritsu Seiyaku Co., Tokyo, Japan) diluted with 0.225 ml saline (Otsuka isotonic sodium chloride solution; Otsuka Pharmaceutical Factory, Inc., Tokushima, Japan). The ovaries of female mice were resected from the retroperitoneum with a vertical incision in the back skin. The testes of male mice were resected from the center of the ventral scrota. The incision sites were closed with metal clips. At 2 weeks after surgery, all mice were treated with i.p. of NNK (2 mg/0.1 ml saline/mouse). After 3 weeks from surgery, Groups 1–4 were treated with another i.p. of NNK (2 mg/0.1 ml saline/mouse). The experiment was terminated after 18 weeks for Groups 1–4 and after 56 weeks for Groups 5–8. All surviving mice were sacrificed under deep anesthesia. Blood samples were collected from the mice in Groups 1–4 to measure the serum concentrations of estradiol and testosterone. These hematological examinations were performed at SRL Inc. (Tokyo, Japan). The detection limit of the estradiol was 10 pg/ml, and that of testosterone was 0.03 ng/ml. Values lower than these limits were considered as 0 and means for each group were calculated. At autopsy, the lungs, livers and kidneys were removed. The lungs were weighed including trachea and heart first, infused with 10% neutral buffered formalin after separation from the trachea and heart, and then immersed in 10% neutral buffered formalin. Lung weights were finally calculated by subtraction of the weight of the remaining trachea and heart. These procedures are appropriate for accurate weighing and good tissue preservation. After fixation in formalin, all lungs were carefully inspected grossly using a stereomicroscope. All macroscopically detected lung nodules were counted and trimmed for histopathological evaluation. The livers and kidneys were weighed and immersed in 10% neutral buffered formalin. Slices of lungs, livers, kidneys and macroscopic mass lesions were routinely processed for embedding in paraffin for histopathological examination of hematoxylin and eosin stained sections.

### Histopathological analysis

Each lung lobe of all mice was examined histopathologically. Lung proliferative lesions were diagnosed as bronchiolo-alveolar hyperplasia, adenoma or adenocarcinoma according to the criteria of ‘International Classification of Rodent Tumors: The Mouse’ (22).

### Statistical analysis

The incidences of lung proliferative lesions (macroscopically and histopathologically) were analyzed by the Fischer’s exact probability test. Body and organ weights were analyzed by the Student’s t-test or the Welch’s t-test between groups. Multiplicities of lung proliferative lesions (macroscopically and histopathologically) were analyzed by the Student’s t-test or the Welch’s t-test between groups. The Student’s t-test was applied when equal variance was obtained and the Welch’s t-test with unequal variance.

## Results

### General conditions

One mouse from Group 3 died 3 weeks after castration. In Group 5, one mouse was sacrificed after 25 weeks from ovariectomy due to poor general condition and another died 54 weeks after ovariectomy. In Group 6, 2 mice were sacrificed 4 and 17 weeks after surgery due to poor general condition and another mouse died 54 weeks after surgery. In addition, one mouse of Group 8 died 53 weeks after surgery. All other mice demonstrated no marked change in their general condition.

### Body and organ weights

Body and organ weights are shown in Table I. In the 18-week study (Groups 1–4), body weights exhibited no significant variation across groups. In the 56-week study (Groups 5–8), body weights in Group 5 (ovariectomy group) were significantly increased as compared with Group 6. Lung weights showed no significant differences between ovariectomy or castration groups and each unoperated group. Liver weights of Group 1 (ovariectomy group) were significantly increased as compared with Group 2, while liver and kidney weights of Group 3 and 7 (castration groups) were significantly decreased as compared with Groups 4 and 8, respectively.

### Sex hormones in the blood samples

Serum concentrations of estradiol and testosterone in each group in the 18-week study (Groups 1–4) are shown in Table II. Blood samples with quantities sufficient for estradiol measurement were obtained from 12 mice of Group 4, and from 9 mice of Groups 1, 2 and 3. For testosterone measurement, 5 mice from Groups 1 and 3, and 8 mice from Groups 2 and 4 were used. In ovariectomized females (Group 1), the estradiol concentration decreased to below the detection limit while the concentration of testosterone was slightly increased compared to Group 2, but without statistical significance. In castrated male mice (Group 3), the concentration of testosterone decreased to below the detection limit compared to Group 4.

### Macroscopical analysis

**Figure 1:**
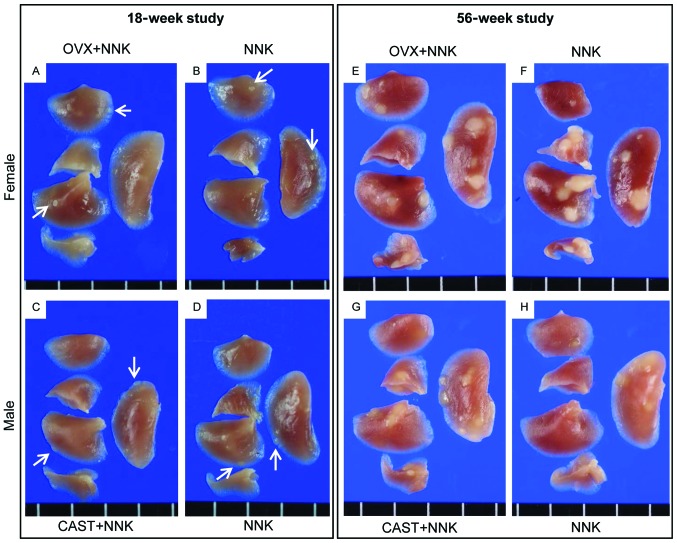
Macroscopical lung nodules. (A-D) In the 18-week study, small white nodules were observed in all groups (arrows indicate representative nodules in Groups 1–4). The sizes of the nodules observed in the 56-week study (E-H) were clearly larger than after 18 weeks (A-D). (A) Female ovariectomized group, Group 1; (B) female unoperated group, Group 2; (C) male castrated group, Group 3; (D) male unoperated group, Group 4; (E) female ovariectomized group, Group 5; (F) female unoperated group, Group 6; (G) male castrated group, Group 7; (H) male unoperated group, Group 8. OVX, ovariectomy; CAST, castration.

Macroscopically, lung white nodules were detected in all mice of all groups (Fig. 1). In Group 7 (56-week study), 2 mice were excluded from macroscopical and histopathological analysis due to the failure in fixation of lungs. Nodules in the 56-week study (Groups 5–8) were clearly larger than those observed after 18 weeks (Groups 1–4). Data for incidences and multiplicities of macroscopical lung nodules are summarized in Table III. The incidences were 100% in all groups. In unoperated groups, the multiplicities of lung nodules were significantly greater in females than in males of both 18- and 56-week studies. In the 56-week study, the multiplicity of lung nodules in the male castrated group (Group 7) was significantly increased as compared to the male unoperated group (Group 8).

### Histopathological analysis

**Figure 2:**
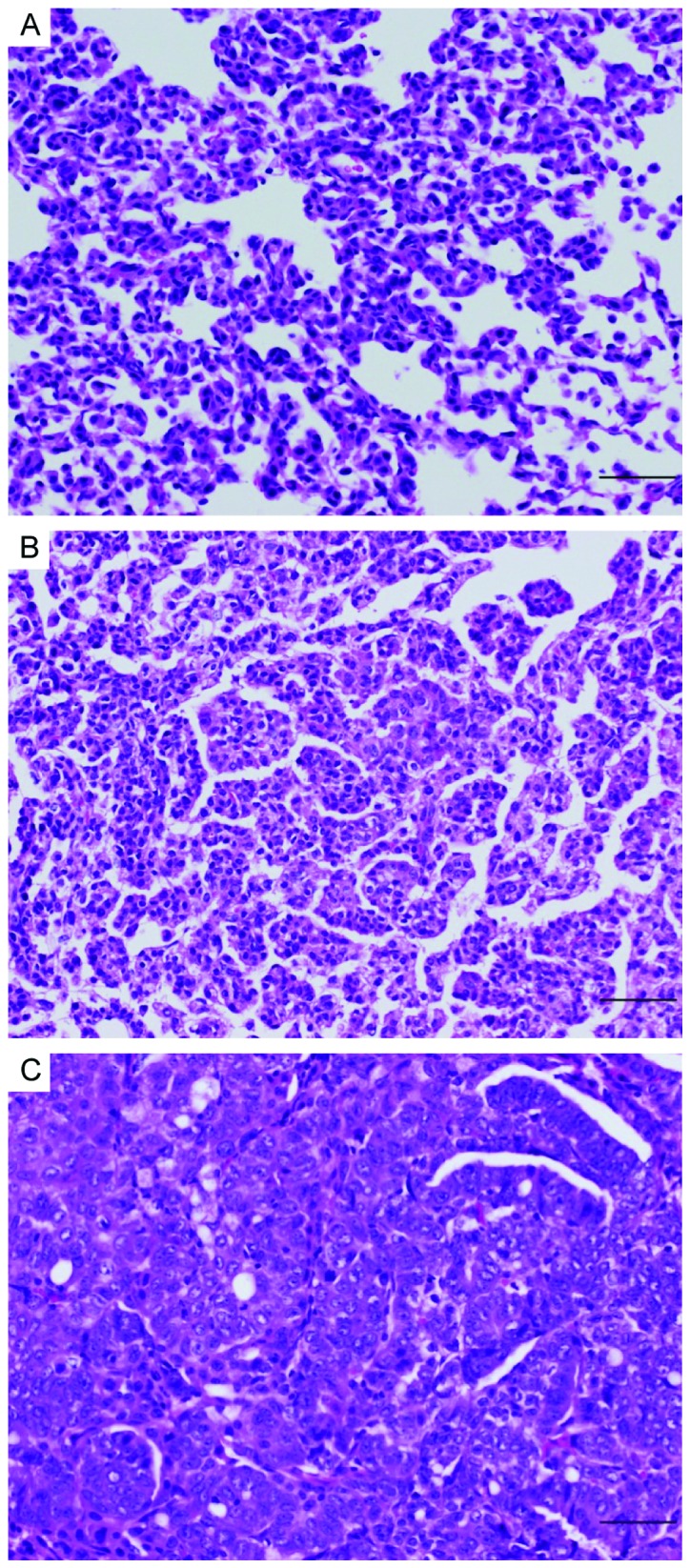
Histopathological lung proliferative lesions. Representative histo- pathology of lung lesions in the 56-week study. Bar, 50 μm. (A) Bronchiolo-alveolar hyperplasia in a Group-7 mouse; (B) adenoma in a Group-6 mouse; (C) adenocarcinoma in a Group-5 mouse.

In histopathological analysis of lung proliferative lesions, bronchiolo-alveolar hyperplasias and adenomas were observed in the 18-week study (Groups 1–4) and bronchiolo-alveolar hyperplasias, adenomas and adenocarcinomas after 56 weeks (Groups 5–8) (Fig. 2). Incidences and multiplicities of each proliferative lesion are summarized in Table IV. The incidences showed no significant intergroup variation in either 18- or 56-week studies. In the 18-week study, the multiplicity of adenomas in the female ovariectomized group (Group 1) was significantly lower than in the female unoperated group (Group 2). In the 56-week study, the multiplicities of hyperplasias, adenomas and tumors (adenomas and adenocarcinomas) in the male castrated group (Group 7) were significantly increased, and carcinomas showed a tendency to increase, compared with the male unoperated group (Group 8). In liver, a hepatocellular carcinoma was observed in only one case of Group 5. No other lesions were observed in liver histopathologically. There were no lesions detected in kidneys in any of the groups. A lipoma surrounding the kidney and a thymoma were observed in Group 5, each at incidences of 1/18 (6%). No other tumors were observed in any of the groups.

## Discussion

In the present 18-week study, the serum concentration of estradiol of female mice was significantly decreased by ovariectomy, while the concentration of testosterone was slightly increased, and the serum concentration of testosterone of male mice was significantly decreased by castration. Estradiol is one of the most important sex steroid hormones secreted by the ovary and testosterone is a principal androgen secreted by the testis (23,24). The decrease with gonadectomy was in line with expectation and the increase in testosterone in ovariectomized females may be due to the secretion by the adrenal cortex. It is known that steroid sex hormones, particularly androgen, are also secreted from adrenal cortex (25), and secretion may be augmented in ovariectomized female mice as a reaction to ovariectomy (26). However, the concentration of testosterone in ovariectomized female mice was much lower than that in unoperated males.

In males, body weights showed no significant difference with castration after both 18 and 56 weeks. In females of the 56-week study, body weights in the operated group (Group 5) were significantly increased and this seemed to be due to the ovariectomy. In ovariectomized female mice, increase of body fat, reduction of lipid metabolism and activation of lipid synthesis have been reported (27–29). In males of the 18- and 56-week studies, the liver and kidney weights of castrated groups (Groups 3 and 7) were significantly decreased compared with the unoperated groups (Groups 4 and 8), and this may be due to the castration, as similar weight loss has been reported in castrated CF-1 mice (30). In females in the 18-week study, the liver weight of the ovariectomized group (Group 1) showed significant increase compared with the unoperated group (Group 2), although no such difference was noted after 56 weeks. As there is no report to support this, the liver weights may be influenced by estradiol derived from the ovary.

In unoperated groups, the multiplicities of lung nodules in females (Groups 2 and 6) were significantly greater than in males (Groups 4 and 8), confirming female A/J mice to be more susceptible to NNK-induced lung carcinogenesis than males. Our results indicated that lung carcinogenesis was increased by castration, suggesting the possibility that testosterone inhibits NNK-induced lung carcinogenesis. In addition, the results of the 18-week study suggest that female sex hormones contribute to the malignant transformation of lung proliferative lesions, such as tumorous alteration from hyperplasia to adenoma. However, the results of the 56-week study did not support this suggestion.

In humans, there have been several reports that sex hormones affect incidences of lung adenocarcinoma. The characteristics of gene mutations in lung cancer are reported to differ between the sexes. In human lung cancer, tobacco-related p53 and EGFR mutations are more common in women than in men (20). Thus, there is a possibility that lung carcinogenesis progresses through different pathways between men and women. Estradiol influences the activity of various metabolic enzymes, such as CYP2A6, CYP1A2, CYP3A4, CYP2C19, UDP-glucuronyltransferase, which can activate NNK to ultimate carcinogenic species (2,31,32). In women, nicotine metabolism by CYP2A6 is reported to be accelerated by estrogen (33). Previously, we demonstrated that CYP2A6 plays important roles in NNK-induced lung carcinogenesis (5–9). In addition, oral contraceptives (estrogen) increase drug metabolism by glucuronidation (34–36). However, there are also reports that oral contraceptives decelerate drug metabolism by CYP1A2, CYP3A4 and CYP2C19 (37–39). Whatever the case, it appears clear that metabolic enzymes of NNK are influenced by estradiol. There is also the possibility that androgen influences lung carcinogenesis. Androgen receptors are present in human lung adenocarcinomas and normal lung tissue of humans and mice, and their expression in normal lung tissue of mice may be affected by castration and testosterone administration (40).

In male mice of the present study, tumors (adenomas and adenocarcinomas) were significantly increased in the castrated group only after 56 weeks, indicating that it is necessary to use long-term experiments in order to determine modifying potential of male sex hormones on lung carcinogenesis. The present study, in fact, pointed to the possibility that NNK-induced lung carcinogenesis may be inhibited by testosterone and accelerated by estradiol, although the effect was only slight in females. One explanation is that lung carcinogenic effects of NNK are so strong in females, that modifying effects of estradiol are masked.

In conclusion, female A/J mice were confirmed to be more susceptible to NNK-induced lung carcinogenesis than males. In males, lung carcinogenesis was increased by castration, whereas in females, malignant transformation of lung proliferative lesions tended to be inhibited by ovariectomy. These results suggested that NNK-induced lung carcinogenesis is inhibited by testosterone and accelerated by estradiol. These findings indicate the possibility that sex hormones play important roles in determining sex differences in lung carcinogenesis in A/J mice initiated by NNK. Additional experiments are ongoing to confirm the effects of the sex hormones themselves.

## Figures and Tables

**Table I:** Body and relative organ weights.

Group	Gender^a^	Treatment^a^	Duration^b^	No.^c^	Body weight^d^ (g)	Lung^d^ Relative (%)	Liver^d^ Relative (%)	Kidneys^d^ Relative (%)
1	F	NNKx2 OVX	18	21	24.81±2.80	0.70±0.08	4.50±0.26^e^	1.10±0.10
2	F	NNKx2 N	18	15	23.76±2.44	0.70±0.08	4.28±0.23	1.13±0.07
3	M	NNKx2 CAST	18	16	26.50±2.67	0.66±0.08	4.22±0.18^f^	0.98±0.05^g^
4	M	NNKx2 N	18	15	27.96±2.20	0.65±0.09	4.61±0.22	1.26±0.11
5	F	NNKx1 OVX	56	18	31.04±4.07^h^	1.23±0.53	4.20±0.52	1.57±2.08
6	F	NNKx1 N	56	12	25.80±3.09	1.27±0.23	4.18±0.76	1.19±0.27
7	M	NNKx1 CAST	56	21	33.19±4.17	0.86±0.21	3.67±0.23^i^	0.80±0.09^j^
8	M	NNKx1 N	56	14	30.33±4.41	0.86±0.18	4.28±0.40	1.34±0.13

a F, female; M, male; OVX, ovariectomy; CAST, castration; N, unoperated.

b Weeks.

c Number of mice examined.

d Means ± standard deviation.

e Significantly different from Group 2 by Student’s t-test (P<0.05).

f Significantly different from Group 4 by Student’s t-test (P<0.001).

g Significantly different from Group 4 by Welch’s t-test (P<0.001).

h Significantly different from Group 6 by Student’s t-test (P<0.05).

i Significantly different from Group 8 by Welch’s t-test (P<0.001).

j Significantly different from Group 8 by Student’s t-test (P<0.001).

**Table II:** Serum concentrations of estradiol and testosterone.

				Estradiol		Testosterone	
					
Group	Gender^a^	Treatment^a^	Duration^b^	No.^c^	(pg/ml)^d^	No.	(ng/ml)^d^
1	F	NNKx2 OVX	18	9	DL^e^	5	0.14±0.1
2	F	NNKx2 N	18	9	19.7±6.9	8	DL
3	M	NNKx2 CAST	18	9	DL	5	DL
4	M	NNKx2 N	18	12	DL	8	1.06±2.4

a F, female; M, male; OVX, ovariectomy; CAST, castration; N, unoperated.

b Weeks.

c Number of mice examined.

d Means ± standard deviation.

e DL, below the detection limit.

**Table III:** Incidences and multiplicities of macroscopical lung nodules.

					Macroscopical nodule	
					
Group	Gender^a^	Treatment^a^	Duration^b^	No.^c^	Incidence (%)	Multiplicity^d^
1	F	NNKx2 OVX	18	21	21/21 (100.0)	17.2±8.4
2	F	NNKx2 N	18	15	15/15 (100.0)	19.7±6.9^e^
3	M	NNKx2 CAST	18	16	16/16 (100.0)	14.3±6.2
4	M	NNKx2 N	18	15	15/15 (100.0)	12.2±5.7
5	F	NNKx1 OVX	56	18	18/18 (100.0)	18.2±7.6
6	F	NNKx1 N	56	12	12/12 (100.0)	20.6±6.0^f^
7	M	NNKx1 CAST	56	19	19/19 (100.0)	12.1±4.6^g^
8	M	NNKx1 N	56	14	14/14 (100.0)	7.6±3.3

a F, female; M, male; OVX, ovariectomy; CAST, castration; N, unoperated.

b Weeks.

c Number of mice examined.

d Means ± standard deviation.

e Significantly different from Group 4 by Welch’s t-test (P<0.05).

f Significantly different from Group 8 by Welch’s t-test (P<0.001).

g Significantly different from Group 8 by Student’s t-test (P<0.05).

**Table IV:** Incidences and multiplicities of lung proliferative lesions.

					Hyperplasia		Adenoma		Adenocarcinoma		Tumor^d^	
								
Group	Gender^a^	Treatment^a^	Duration^b^	No.^c^	Incidence (%)	Multiplicity^e^	Incidence	Multiplicity	Incidence	Multiplicity	Incidence	Multiplicity
1	F	NNKx2 OVX	18	21	21/21 (100.0)	4.8±3.6	18/21 (85.7)	3.1±2.3^f^	0/21 (0.0)	0	-	-
2	F	NNKx2 N	18	15	14/15 (93.3)	3.7±2.4	15/15 (100.0)	5.8±3.7	0/15 (0.0)	0	-	-
3	M	NNKx2 CAST	18	16	16/16 (100.0)	5.3±2.9	14/16 (87.5)	2.9±2.8	0/15 (0.0)	0	-	-
4	M	NNKx2 N	18	15	13/15 (86.7)	4.1±2.7	14/15 (93.3)	3.3±2.0	0/15 (0.0)	0	-	-
5	F	NNKx1 OVX	56	18	17/18 (94.4)	5.3±3.5	18/18 (100.0)	9.7±3.8	9/18 (50.0)	0.9±1.0	18/18 (100.0)	10.6±4.2
6	F	NNKx1 N	56	12	11/12 (91.7)	4.8±2.7	12/12 (100.0)	10.2±4.6	7/12 (58.3)	0.8±0.8	12/12 (100.0)	11.0±4.5
7	M	NNKx1 CAST	56	19	19/19 (100.0)	4.5±2.2^g^	19/19 (100.0)	4.9±3.7^g^	9/19 (47.4)	0.7±1.1	19/19 (100.0)	5.6±4.0^g^
8	M	NNKx1 N	56	14	14/14 (100.0)	3.1±1.1	12/14 (85.7)	2.5±1.7	4/14 (28.6)	0.3±0.5	12/14 (85.7)	2.8±2.0

a F, female; M, male; OVX, ovariectomy; CAST, castration; N, unoperated.

b Weeks.

c Number of mice examined.

d Adenoma and adenocarcinoma.

e Means ± standard deviation.

f Significantly different from Group 2 by Welch’s t-test (P<0.05).

g Significantly different from Group 8 by Welch’s t-test (P<0.05).
